# L-thyroxine Stabilizes Autoimmune Inflammatory Process in Euthyroid Nongoitrous Children with Hashimoto’s Thyroiditis and Type 1 Diabetes Mellitus

**DOI:** 10.4274/Jcrpe.1136

**Published:** 2013-12-12

**Authors:** Katarzyna Korzeniowska, Przemyslawa Jarosz-Chobot, Agnieszka Szypowska, Anna Ramotowska, Wojciech Fendler, Barbara Kalina-Faska, Agnieszka Szadkowska, Wojciech Mlynarski, Malgorzata Mysliwiec

**Affiliations:** 1 Medical University of Gdansk, Department of Pediatrics, Diabetology and Endocrinology, Gdansk, Poland; 2 Medical University of Silesia, Department of Pediatrics, Endocrinology and Diabetes, Silesia, Poland; 3 Medical University of Warsaw, Department of Diabetology, Newborn Pathology and Birth Defects, Warsaw, Poland; 4 Medical University of Lodz, Department of Pediatrics, Oncology, Hematology and Diabetology, Lodz, Poland

**Keywords:** autoimmune thyroiditis, type 1 diabetes mellitus, L-thyroxine treatment, lipid profile, glycemic control, thyroid volume SDS

## Abstract

**Objective: **To investigate if L-thyroxine (T4) treatment may influence the clinical course of autoimmune thyroiditis (AIT) or prevent progression to subclinical or overt hypothyroidism in euthyroid nongoitrous pediatric patients with type 1 diabetes mellitus (T1DM) and AIT.

**Methods:** The study was performed in four Polish pediatric diabetes centers. Of 330 children with T1DM and AIT followed between 2008 and 2012, 101 received L-T4 and 160 underwent clinical observation for 24 months. Thyroid stimulating hormone (TSH), free T4 (fT4), anti thyroid peroxidase antibody (anti-TPO), anti thyroglobulin antibody (anti-TG), glycosylated hemoglobin (HbA1c) levels, and lipid profile were assessed in all patients. Ultrasonographic evaluation was also performed in all children at each examination.

**Results:** Patients treated with thyroid hormones had higher TSH levels (3.99; interquantile 3.5 to 4.52 vs. 2.09 mIU/L; interquantile 1.55 to 3.06; p<0.0001). A fall in TSH level (0.87 mIU/L 95% CI 0.43-1.30; p<0.0001) was documented after the first year of treatment. FT4 level did not differ between the groups at baseline (p=0.7434), but rose in the treatment group and fell in the control group [mean difference 0.78 95% CI

-0.22-1.53 pmol/L (p=0.02) after 12 months and 0.98 95% CI 0.04-1.76 (p=0.005) after 24 months]. Higher levels of anti-TPO were initially found in the treated patients (p<0.0001) and significantly decreased over the 24-month period (p<0.0001). Children in the treatment group had higher anti-TG levels (p<0.0001), which showed a borderline decrease (p=0.08) in time. In the control group, anti-TG levels rose marginally (p=0.06) during the study.

**Conclusions:** The data demonstrate that treatment with L-T4 in euthyroid pediatric patients with T1DM and AIT stabilizes autoimmune inflammation in the thyroid gland and is to be recommended as soon as the diagnosis is established.

**Conflict of interest:**None declared.

## INTRODUCTION

Autoimmune thyroiditis (AIT) is the most common disease accompanying type 1 diabetes mellitus (T1DM) in children. To date, there are still no commonly accepted guidelines for the screening and management of Hashimoto’s disease in patients with T1DM. International Society for Pediatric and Adolescent Diabetes Consensus 2009 (1) suggests estimation of thyroid stimulating hormone (TSH), free thyroxine (fT4), and thyroid antibodies at the time of diagnosis of T1DM and then, if there are no signs of thyroid dysfunction, to continue this process with 2-year intervals. According to the American Diabetes Association 2012 Guidelines (2), patients with newly diagnosed diabetes mellitus should be screened for AIT after metabolic stabilization and subsequently, the patient should be checked for thyroid gland enlargement and decrease in growth velocity at follow-up visits every 12-24 months. Diabetes Poland (3), on the other hand, recommends annual screening for AIT in children suffering from T1DM. Systematic screening implementation guarantees early recognition and adequate treatment of thyroid dysfunction which may protect against further deterioration of thyroid function.

The other clinical problem is a lack of agreement among various International Diabetes Associations on time of initiation of treatment. Most clinicians agree on starting treatment in patients with AIT if the TSH level is higher than 10 mIU/L because of significant risk of overt hypothyroidism (4). However, there is still no consensus on whether treatment with L-T4 should start from the beginning of AIT or only when TSH level is elevated (5).

The aim of our study was to investigate whether L-T4 treatment has an influence on the autoimmune inflammatory process in the thyroid gland and also to assess its effect on metabolic control and lipid profile in euthyroid nongoitrous children with autoimmune poliglandular syndrome type 3a. 

## METHODS

We retrospectively analyzed the medical records of 330 pediatric patients with T1DM and AIT who had been treated in four academic regional diabetes centers in Poland (Warsaw, Katowice, Lodz and Gdansk) between 01.01.2008 and 31.12.2012. These four centers have treated 38% of children with diabetes in Poland and have previously collaborated in large-scale epidemiologic studies.

All children in the study were euthyroid (TSH level under 5 mIU/L, fT4 in normal range) and without goiter [thyroid volume standard deviation score (SDS) were calculated with reference to ultrasonographic (US) findings in healthy Polish children] (6). Patients with any autoimmunological disease other than T1DM and AIT were excluded from the study.

The diagnosis of Hashimoto’s disease was based on US findings (volume, hypoechogenicity, lymph nodes), biochemical markers including TSH, fT4 levels, anti-thyroglobulin (anti-TG) or/and anti-thyroid peroxidase (anti-TPO) antibody titers. The patients were divided into two groups: 101 were treated with L-T4 for two years (treatment was started with 100

µg/m2/day). These patients had TSH levels higher than 3

mIU/mL, their antibody levels were above the normal range, or their US results revealed hypoechogenicity in over 50% of the thyroid gland, and these patients had clinical or biochemical signs of hypothyroidism; 229 children who did not meet these criteria for L-T4 treatment were also followed for two years. During the 24-month observation period of this second group of patients, 69 (31 after 12 and 38 after 24 months) developed subclinical or overt hypothyroidism and started receiving L-T4 therapy. The results on these 69 patients were not included in the statistical analyses. Thus, we analyzed the data of 261 patients: 101 treated with L-T4 (treatment group) and 160 who underwent only clinical observation (control group). All patients underwent clinical and US examination of the thyroid at the beginning of the study and at the 12th and 24th months of the follow-up. US was performed using ACUSON X3000 with 5-7.5 MHz linear compound scanner. Serum samples were drawn at the same time points for measurement of TSH, fT4, anti-TPO, anti-TG, glycosylated hemoglobin (HbA1c), total cholesterol, high-density lipoprotein cholesterol, low-density lipoprotein cholesterol, triglycerides. TSH (normal range 0.35-4.94 mIU/L) and fT4 (normal range 896-18.94 pmol/L) were analyzed by CMIA (Abbott, Wiesbaden, Germany). Serum Anti-TPO (normal range <5.61 IU/mL) and serum anti-TG (normal range <4.11 IU/mL) were determined by CMIA (Abbott, Wiesbaden, Germany). HbA1c assays were performed by ion-exchange HPLC (Variant HbA1c Program; Bio-Rad Laboratories, Hercules, CA, USA). The method used has been certified by the National Glycohemoglobin Standardization Program (NGSP) (http://www.ngsp.org/docs/methods.pdf; last accessed 23 November 2012). Reference values for healthy people estimated by the local laboratory ranged from 4.3 to 5.7% (35-42 mmol/mol). Lipid profile was analyzed by ARCHITECT cSystem and AEROSET, Abbott, Wiesbaden, Germany.

The study proposal was approved by an independent ethics committee, the Ethics Committee of the Medical University of Gdansk, Poland (NKEBM319/2010). Reported investigations have been carried out in accordance with the principles of the Declaration of Helsinki as revised in 2000.

**Statistical Analysis**

The results are presented as medians with 95% CI. General linear regression models with effects analysis were used for multivariate analysis of time- and treatment-dependent effects. Post-hoc comparisons between subgroups were performed using the Tukey’s test. P-values of lower than 0.05 were considered significant.

## RESULTS

Patients receiving L-T4 treatment and the controls had comparable diabetes duration, body mass index (BMI) SDS, glycemic control (HbA1c), fT4 level, thyroid volume SDS and lipid profile, while TSH levels and thyroid antibodies titers were higher in the treatment group (Table 1).

No statistically significant differences were found between the treatment and control groups in their pubertal status by Tanner stages. Chronological age was slightly higher in the patients treated with L-T4 (Table 2).

**Figure 1:**
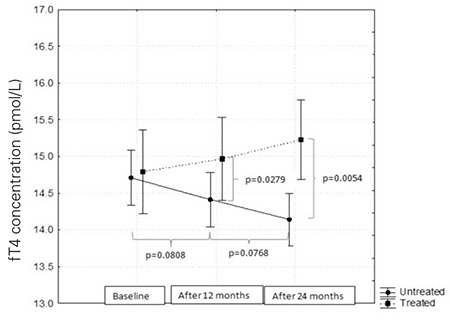
Free thyroxine (fT4) levels in the treatment and control groups

After 12 and 24 months, fT4 level was found to increase in patients treated with thyroid hormones and to gradually decrease in the control group. Differences between the groups were statistically significant (p=0.02 and p=0.005, respectively) (Figure 1).

**Figure 2:**
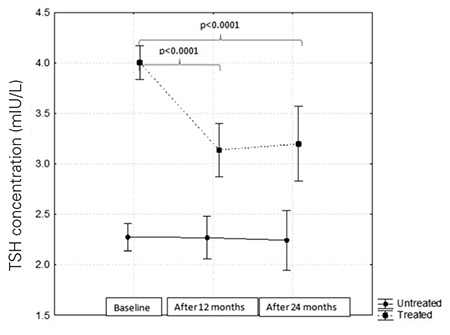
Thyroid stimulating hormone (TSH) levels at the beginning and after 12 and 24 months of follow-up

TSH levels in children treated with L-T4 were high as compared to the controls (F=88.14; p<0.0001). After one year of hormone substitution, a fall in TSH level and its stabilization within the next 12 months was documented. In the controls, the TSH level did not change during the two years of follow-up (Figure 2).

No differences between the treatment and control groups were found in lipid profile, thyroid volume SDS, HbA1c level, or BMI SDS.

**Figure 3:**
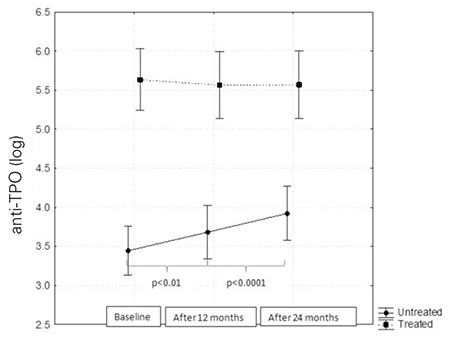
Anti thyroid peroxidase antibody (anti-TPO) levels before and

In patients treated with L-T4, higher levels of anti-TPO were found (by 8.94 95% CI 2.87-39.45; p<0.0001) and the anti-TPO levels were maintained at the same level throughout the study, while anti-TPO levels which were lower in the control group as compared to the treatment group initially, were found to show a systematic increase (p<0.0001) (Figure 3).

**Figure 4:**
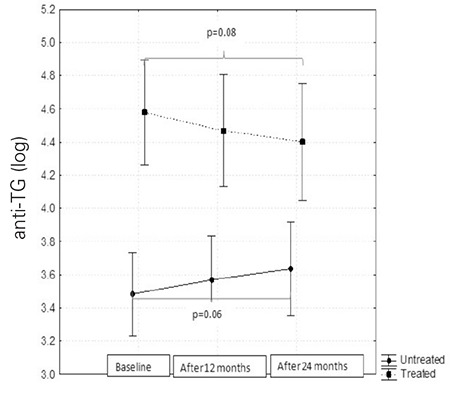
Anti thyroglobulin antibody (anti-TG) levels before and after 12 and 24 months of follow-up

The children in the treatment group had higher anti-TG levels initially (by 2.99 95% CI 1.51-5.09; p<0.0001) and slowly decreased (p=0.08), while these levels showed a gradual increase in the controls (p=0.06). These differences did not reach statistical significance (Figure 4).

## DISCUSSION

The data of this study show that treatment of euthyroid nongoitrous patients with T1DM and AIT leads to stabilization of anti-TPO levels and decrease of anti-TG titers and thus, with an increase of fT4 levels, protects the thyroid gland against further deterioration of thyroid function. Furthermore, subclinical or overt hypothyroidism occurred in 20% (69/330) of the children in our control group, a finding which suggests a higher risk of thyroid dysfunction progression in patients with autoimmune poliglandular syndrome type 3a than in the general pediatric population (7,8,9). According to one hypothesis, T4 administration causes a decrease in TSH level and this may indirectly lead to a decrease in magnitude of antigenic presentation (10). On the other hand, some studies on animal models support the thesis that T4 itself, by direct modulatory effects, may cause a stabilization of the autoimmunity (11,12). Gupta et al (11) after L-T4 treatment in mice, reported increased viability of skin grafts, decreased primary antibody concentrations and inhibition of cellular immune response (11).

Substitution of thyroid hormones is obligatory in hypothyroid patients with AIT (13). However, treatment of euthyroid patients without goiter is still controversial. At present, data evaluating thyroid hormone substitution in children with T1DM are scarce. Kordonouri et al (14) investigated the effect of L-T4 treatment in 15 goitrous euthyroid diabetic patients and reported a significant decrease in thyroid volume SDS only in the treatment group, while an insignificant enlargement of the thyroid gland was noted in the patients without replacement therapy. On the other hand, Padberg et al (15), in a randomized prospective study on 21 euthyroid nongoitrous patients with Hashimoto’s disease, did not find any significant changes in thyroid volume SDS between treatment and control groups at baseline and after 12 months of replacement therapy.

Contradictory data were reported where thyroid antibodies are concerned. Although some clinical studies found a decrease of anti-TPO titers in patients treated with L-T4, (15,16,17), others did not confirm these correlations (18,19). In patients without hormonal substitution, a gradual but not statistically significant increase of anti-TPO titers was reported (15,17,20). Anti-TG titers also changed as a result of L-T4 treatment and most studies reported a gradual decrease in anti-TG titers in treatment groups, whereas in children without hormonal substitution, anti-TG antibodies titers showed an increase during the follow-up (15).

The natural course of AIT in children has been of huge recent interest among investigators. The recent metaanalysis of Cappa et al (21) confirmed that TSH levels, although fluctuating throughout observations, showed a tendency to gradually increase over time, leading to thyroid dysfunction. Kordonouri, Fendler and Jarosz-Chobot et al (22,23,24) followed 126 children with positive thyroid antibodies titers for 5 years and claimed that before maturity, approximately 14% of children with T1DM and AIT required L-T4 treatment because of subclinical or overt hypothyroidism.

To sum up, our findings also indicate that the treatment of patients with autoimmune polyglandular syndrome type 3a who are euthyroid or subclinically hypothyroid is to be recommended. This treatment needs to be started as soon as Hashimoto’s disease is diagnosed.

## Figures and Tables

**Table 1:**
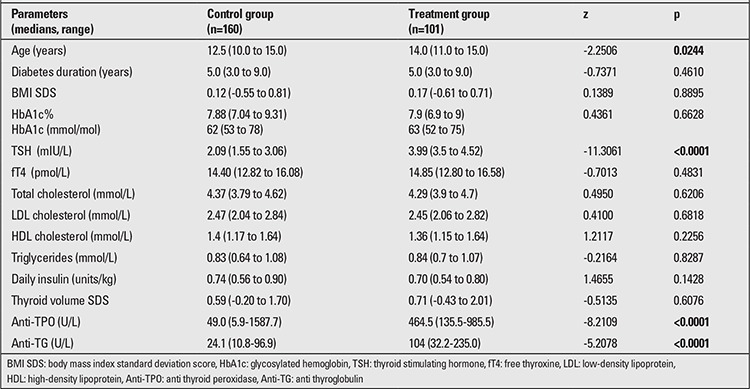
Findings in the treatment and control groups at the beginning of the study

**Table 2:**
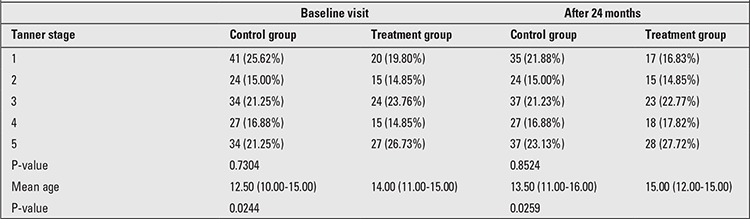
Age and Tanner stage of treatment group and control group of the study
